# Associations between estradiol and hyperuricemia and the mediating effects of TC, TG, and TyG: NHANES 2013–2016

**DOI:** 10.3389/fendo.2024.1422470

**Published:** 2024-08-07

**Authors:** Chuxin Zhang, Hongyang Qian, Yiwei Cui, Xiaojuan Li, Yuli Cheng, Lin Gao

**Affiliations:** ^1^ School of Traditional Chinese Medicine, Beijing University of Chinese Medicine, Beijing, China; ^2^ Qi-Huang Chinese Medicine School, Beijing University of Chinese Medicine, Beijing, China

**Keywords:** estradiol, hyperuricemia, triglyceride, cholesterol, NHANES

## Abstract

**Objectives:**

To explore the relationship between estradiol (E2) and the incidence of hyperuricemia (HUA) in adult women and to explore whether glucolipid metabolism disorders play a mediating role in mediating this relationship.

**Methods:**

A total of 2,941 participants aged 20–65 years were included in the National Health and Nutrition Examination Survey (NHANES) 2013–2016. Multivariate logistic regression analysis was performed to evaluate the correlations of E2 with HUA. Multivariate linear regression analysis was performed to evaluate the associations between E2 and triglyceride (TG), total cholesterol (TC), and the triglyceride-glucose index (TyG). The restricted cubic spline (RCS) model was used to further explore the association between E2 and HUA and between TG, TC, and TyG and HUA. Mediation analyses were performed to examine whether TC, TG, and TyG mediated the relationship between E2 and HUA.

**Results:**

After adjusting for covariates, logistic regression revealed that ln(E2) was significantly associated with HUA in the female subgroup (*p* = 0.035) and that the incidence of HUA tended to increase with decreasing ln(E2) (*p* for trend = 0.026). Linear regression showed that E2 was significantly associated with TC (*p* = 0.032), TG (*p* = 0.019), and TyG (*p* = 0.048). The RCS model showed that ln(E2) was linearly correlated with the incidence of HUA (*p*-overall = 0.0106, *p*-non-linear = 0.3030). TC and TyG were linearly correlated with HUA (TC: *p*-overall = 0.0039, *p*-non-linear = 0.4774; TyG: *p*-overall = 0.0082, *p*-non-linear = 0.0663), whereas TG was non-linearly correlated with HUA. Mediation analyses revealed that TC, TG, and TyG significantly mediated the relationship between ln(E2) and HUA (TC, indirect effect: −0.00148, 7.5%, *p* = 0.008; TG, indirect effect: −0.00062, 3.1%, *p* = 0.004; TyG, indirect effect: −0.00113, 5.6%, *p* = 0.016).

**Conclusion:**

In conclusion, this study demonstrated that compared with women aged 20–45 years, women aged 45–55 years and 55–65 years had lower E2 levels and a greater incidence of HUA. E2 levels and the incidence of HUA were negatively associated in female individuals but not in male individuals. In addition, TC, TG, and TyG, which are markers of glucolipid metabolism, played a mediating role in the association between E2 and HUA.

## Introduction

Uric acid (UA) is the end product of purine metabolism in the liver, intestine, and other tissues (such as muscle and kidney). Abnormally high levels of serum uric acid (SUA), known as hyperuricemia (HUA), are involved in the occurrence and development of many metabolic diseases, cardiovascular diseases, and chronic kidney diseases (1). It has been reported to be an independent risk factor for type II diabetes, hypertension, chronic kidney disease, gout, etc. (2–5), and poses a great threat to health. Previous studies have shown that the prevalence of HUA in the overall population is 10.3% to 20.2% in different countries and regions (6–8). Unfortunately, it has significantly increased in recent years.

Factors related to SUA levels, which we are familiar with, include diet, alcohol consumption, obesity, and genetic polymorphisms (1). In addition, numerous studies have shown that uric acid levels in women are linked to periods of menstruation status. SUA levels in perimenopausal and postmenopausal women showed an increasing trend, and the incidence of HUA was also significantly greater than that in young women (9–11). The continuous decrease in estradiol (E2) during perimenopause and postmenopause is considered to be one of the reasons for this phenomenon (12, 13). However, the effect of E2 on SUA levels is not widely recognized. Compared with other risk factors for HUA, the contribution of menopause to the prevalence of HUA seems to be not widely known. Therefore, this population is particularly relevant to a broader understanding of HUA. This may promote women to take timely measures to reduce HUA and its related consequences.

It is widely known that a reduction in E2 in women during perimenopause and postmenopause can cause lipid and glucose metabolism disorders and increase the risk of cardiovascular disease (14–16). Dysfunction of glucolipid metabolism is considered to be a risk factor for elevated SUA levels. Increases in triglycerides (TG), total cholesterol (TC), and the triglyceride-glucose index (TyG), which are indicators related to glucolipid metabolism disorders, have been reported to be related to an increased incidence of HUA (17–19). In addition, UA is closely related to cardiovascular disease. It was found to be an independent predictor of cardiovascular mortality (20), which is associated with the occurrence of cardiovascular diseases such as atherosclerosis, acute coronary syndrome, and heart failure (HF) (21–23). A recent study on SUA and long-term prognosis in HF patients showed that SUA was an independent prognostic predictor of long-term outcome in HF in men, but not in women (24). This study suggested that for women, there may be other factors closely related to sex involved in the interaction of UA metabolism with glucolipid metabolism and related diseases.

For perimenopausal and postmenopausal women, it is largely unknown whether the prevalence of HUA is affected by changes in other metabolic indicators during a reduction in E2 levels. Previous studies have reported changes in UA levels during the perimenopausal period and the relationship between glucolipid metabolism and UA. The impact of changes in E2 levels on glucolipid metabolism and subsequent cardiovascular hazards has always been a topic of constant concern. However, it is not clear whether glucolipid metabolism plays a certain role between E2 changes and UA levels, which is the focus of this study. Therefore, the purpose of this study was to explore the relationship between E2 and the prevalence of HUA in adult women and to explore whether glucolipid metabolism disorders play a mediating role in this relationship.

## Methods

### Study population

**Figure 1:**
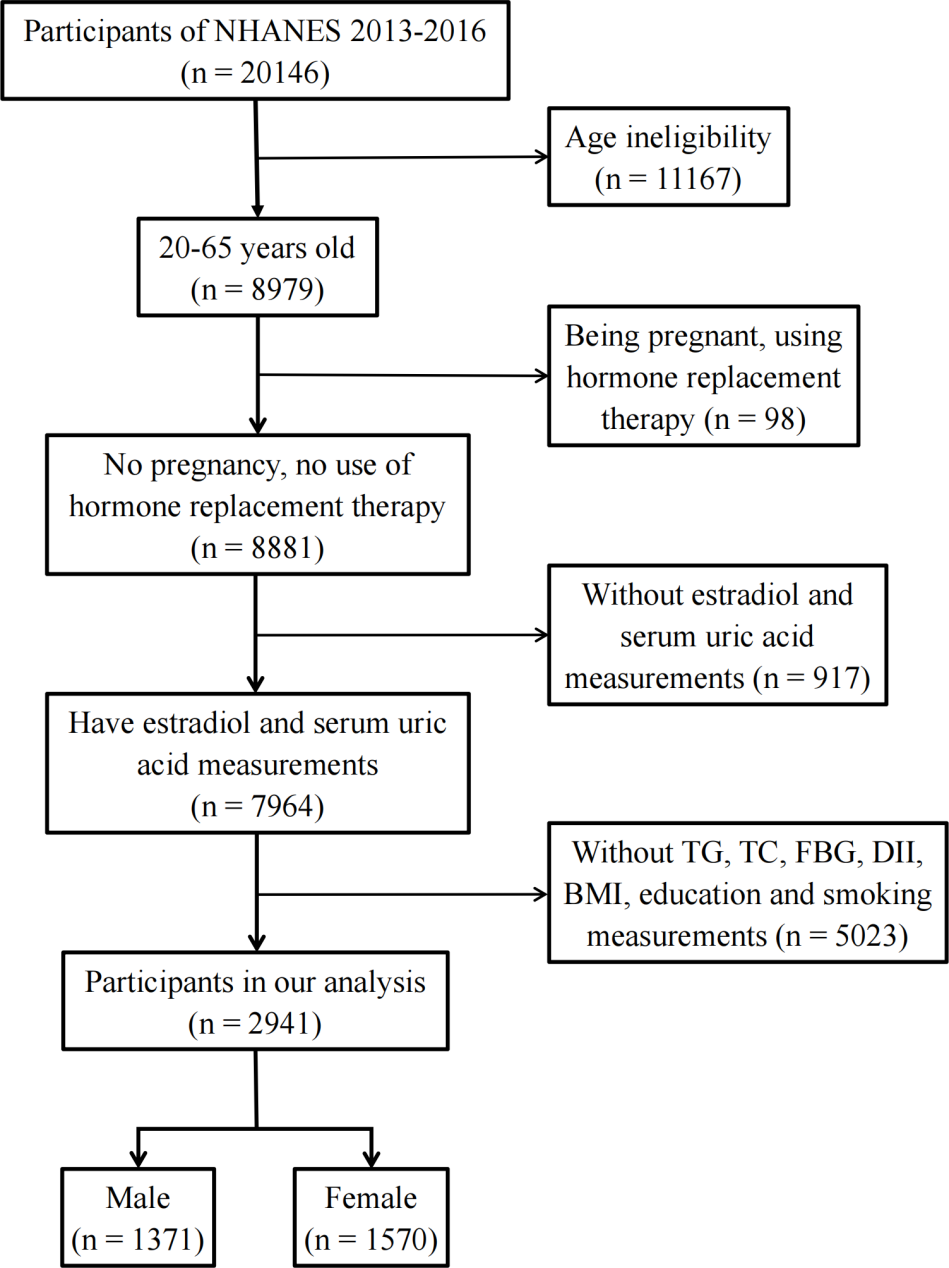
Flow diagram for selecting eligible participants from the NHANES.

The National Health and Nutrition Examination Survey (NHANES) is an ongoing cross-sectional survey of representative samples of the civilian non-institutionalized U.S. population conducted by the Centers for Disease Control and Prevention (CDC)’s National Center for Health Statistics (NCHS). Study participants were sampled through a complex multistage probability sampling design. The data were deidentified by the NCHS before publication. Ethical approval was not needed for this study because the study was based on secondary analyses of publicly available data. The datasets generated and analyzed in the present study are readily available on the official NHANES website (https://www.cdc.gov/nchs/nhanes/index.htm). Among the 11 NHANES cycles from 1999 to 2020, only two NHANES cycles (2013–2014 and 2015–2016) reported individual E2 levels Therefore, these two NHANES cycles were included in this study to obtain enough data on E2. To exclude individuals with incomplete data, participants aged 20–65 years with complete data on E2 levels, uric acid concentrations, fasting blood glucose (FBG) levels, body mass index (BMI), dietary inflammatory index (DII), smoking status, and education level were enrolled in this study. In addition, hormone replacement therapy can mask an individual’s true hormone levels. Pregnancy causes physiological changes in women’s sex hormone levels. Therefore, individuals who had used hormone replacement therapy (“taking estrogen only pills now?”, “taking progestin-only pills now?”, “using estrogen-only patches now?”, and “using estrogen/progestin patches now?”) or who were pregnant (“are you pregnant now?”) were excluded (**Figure 1**).

### Assessment of exposure: E2

E2 concentrations were determined by isotope dilution chromatography-tandem mass spectrometry (ID-LC−MS/MS), which was developed by the CDC. The lower limit of E2 detection was 2.994 pg/mL. Values less than the lower limit are replaced by the lower limit of detection divided by the square root of 2 (25).

### Assessment of outcome: HUA

The uric acid concentration was detected on a Beckman Synchron LX20 (Beckman Coulter, Inc., Brea, CA) using a colorimetric method. According to the NHANES website, these data do not indicate the existence of a low limit of detection, so there is no need to convert the lower limit value (26). HUA was defined as a uric acid concentration greater than 6 mg/dL in female participants and 7 mg/dL in male participants (12). In addition, in a recent study of the Uric Acid Right for Heart Health (URRAH) Project, a uric acid threshold (UAT) that is closely related to cardiovascular disease outcomes was reported, which is lower than the clinical HUA diagnostic criteria (27). UAT was defined as a uric acid concentration greater than 5.1 mg/dL in female participants and 5.6 mg/dL in male participants, as the study reported. By comparing the existing clinical criteria with the uric acid threshold, which is more suitable for cardiovascular status, we wanted to determine whether hyperuricemia caused by changes in hormone levels in women during perimenopause may be associated with cardiovascular risk. Moreover, whether the relationship between the E2 level and UA level may differ due to different UA cut-off values is unclear.

### Assessment of mediators: TG, TC, and TyG levels

TG was detected on a Roche modular P and a Roche Cobas 6000 chemical analyzer using a colorimetric method. TC was measured by an enzymatic method on a Roche modular P and a Roche Cobas 6000 chemical analyzer. The FBG was measured with a Roche C501 and C311 instrument using an enzymatic method. The calculation formula for TyG was as follows: TyG = ln[TG (mg/dL)×FBG (mg/dL)/2] (28). In the data description of the NHANES website, these data do not report the existence of a low limit of detection, so there is no need to convert the lower limit value (29, 30).

### Covariates

Continuous covariates included age, DII, and BMI. Participants’ dietary intake was assessed using two previously validated 24-h dietary recalls (24DRs). The DII, which was shown to be associated with increased HUA risk in a previous study (31), was calculated from 28 foods provided by the 24DR with the R package “dietaryindex” (32); the DII included information on energy, protein, carbohydrate, dietary fiber, total fatty acid, total saturated fatty acid, monounsaturated fatty acids (MUFAs), polyunsaturated fatty acids (PUFAs), cholesterol, β-carotene, niacin, folate, magnesium, iron, zinc, selenium, caffeine, alcohol, n3 polyunsaturated fatty acid, n6 polyunsaturated fatty acid, and vitamins A, B1, B2, B6, B12, C, D, and E (33). BMI was calculated as weight in kilograms divided by the square of height in meters.

Gender (male/female), race (Mexican American, other Hispanic, non-Hispanic White, non-Hispanic black, or other race), poverty income ratio (PIR) (poor/not poor), education level (<high school/completed high school/>high school), marital status (married/living with partner, or widowed/divorced/separated/never married), alcohol status (yes/no), smoking status (never/former/current), physical activity (inactive/moderate/vigorous/both moderate and vigorous), cancer (yes/no), chronic kidney disease (CKD) (yes/no), diabetes (yes/no), hypertension (yes/no), and liver disease (yes/no) were used as categorical variables.

Alcohol status was obtained from two 24-h dietary recalls, and participants were defined as alcohol users if they reported alcohol consumption in at least one 24-h dietary recall. Smoking status was assessed as never smoked (smoking <100 cigarettes), former smoking (not currently smoking but smoking ≥100 cigarettes), or current smoking (≥100 cigarettes and currently smoking every day or on some days). Physical activity was assessed by vigorous physical activity (high-intensity activities, fitness, and sports such as running or basketball) and moderate physical activity (e.g., brisk walking, swimming, and bicycling at a regular pace). Cancer and liver disease were diagnosed by self-reports (“ever told you had cancer or malignancy” and “ever told you had any liver condition”).

There is an interaction between UA and kidney disease, so it is necessary to include CKD as one of the covariates (34, 35). The eGFR was determined through the application of the Chronic Kidney Disease Epidemiology Collaboration creatinine equation. For men, the equation was as follows: eGFR=(140-age)×body weight (kg)×1.23/creatinine (mmol/L). For women, the equation was eGFR=(140-age)×body weight (kg)×1.03/creatinine (mmol/L) (31). CKD was diagnosed when the eGFR was < 60 mL/(min·1.73 m^2^) or when the urine albumin creatine ratio (UACR) was ≥30 mg/g (36, 37).

Diabetes was diagnosed when patients met one or more of the following criteria: ① had a medical diagnosis of diabetes (“doctor told you have diabetes”), ② were taking antidiabetic drugs (“taking insulin now” or “taking diabetic pills to lower blood sugar”), ③ had a glycohemoglobin A1c (HbA1c) level > 6.5%, or (4) had an FBG ≥ 7.0 mmol/L (126 mg/dL) (38).

Three or four consecutive blood pressure levels were obtained for every participant. The mean of all available measurements was used to define the systolic blood pressure (SBP) and diastolic blood pressure (DBP) levels. Hypertension was diagnosed when patients met one or more of the following criteria: ① a medical diagnosis of hypertension (“ever told you have high blood pressure”), ② an SBP ≥140 mmHg, or ③ a DBP ≥90 mmHg (39).

### Statistical analysis

The statistical software R 4.3.2 was used for statistical analysis. A two-tailed p value <0.05 was considered to indicate statistical significance in all analyses. To understand the overall situation of the population, descriptive statistics were performed using the R package “compareGroups” (40). Continuous variables are expressed as the means and standard deviations or medians and interquartile ranges, and categorical variables are expressed as proportions and percentages of the total. The χ^2^ test was used to compare categorical variables between groups. For continuous variables, one-way analysis of variance was used to compare normally distributed variables, and the Kruskal−Wallis test was used to compare variables with a skewed distribution between groups.

We hypothesized that E2 levels are associated with the prevalence of TG, TC, TyG, and HUA. To verify this, multivariate logistic regression analysis was performed to evaluate the correlations of E2 with HUA, whereas multivariate linear regression analysis was performed to evaluate the associations between E2 and TG, TC, and TyG. The R packages “survey” and “gtsummary” (41) were used.

Based on the results of the regression analysis above, the trend in the E2 quintile with HUA was evaluated simultaneously. In this way, it was further clarified whether there were differences in the correlation between E2 and HUA incidence in different strata.

To determine whether there was a non-linear relationship between the indicators and to avoid overfitting, the restricted cubic spline (RCS) model was used to further explore the association between E2 and HUA and the association between TG, TC, TyG, and HUA with the R package “rms”.

Finally, we hypothesized that the glucolipid metabolism indicators TC, TG, and TyG are involved in mediating the relationship between E2 levels and the incidence of HUA. To test this hypothesis, mediation analyses were performed with the R package “mediation”. The size of the indirect pathway effect, the proportion of the mediating effect, and the p value for the mediating effect are all shown in the results. The levels of E2 were transformed into natural logarithms, ln(E2), in all analyses, and the raw values are presented for descriptive purposes only. Unweighted models were used in mediation analyses, whereas weighted models were used in the RCS model. Multivariate logistic/linear regression was performed with both unweighted models and weighted models.

## Results

### Participant characteristics

Eventually, 2,941 participants were included in this cross-sectional study (**Figure 1**). We divided the participants into male (n = 1,371) and female (n = 1,570) groups and further categorized them by age into 20–45 years, 45–55 years, and 55–65 years (**Table 1**). The data showed that 20.1% of the male participants and 13.4% of the female participants had HUA. In the female population, the average E2 level of women aged 20–45 years was 64.6 pg/mL, and the 25%–75% fluctuation range was 32.1 pg/mL–131 pg/mL. The average E2 level of women aged 45–55 years was 18.2 pg/mL, and the 25%–75% fluctuation range was 6.24 pg/mL–94.8 pg/mL. The average E2 level of women aged 55–65 years was 6.27 pg/mL, and the 25%–75% fluctuation range was 3.3 pg/mL–10.9 pg/mL. The median E2, TC, TG, and TyG levels were 31.0 pg/mL, 187 mg/dL, 83.0 mg/dL, and 9.13, respectively, in female participants and 25.3 pg/mL, 189 mg/dL, 100 mg/dL, and 9.18, respectively, in male participants.

**Table 1:** Characteristics of the study participants.

Age	Male					Female				
	ALL	20–45	45–55	55–65	*p*.overall	ALL	20–45	45–55	55–65	*p*.overall
	N=1371	N=691	N=300	N=380		N=1570	N=815	N=342	N=413	
Education level:					0.083					0.847
<High school	87 (6.35%)	32 (4.63%)	25 (8.33%)	30 (7.89%)		105 (6.69%)	52 (6.38%)	25 (7.31%)	28 (6.78%)	
Completed high school	189 (13.8%)	98 (14.2%)	35 (11.7%)	56 (14.7%)		180 (11.5%)	92 (11.3%)	44 (12.9%)	44 (10.7%)	
>High school	1,095 (79.9%)	561 (81.2%)	240 (80.0%)	294 (77.4%)		1,285 (81.8%)	671 (82.3%)	273 (79.8%)	341 (82.6%)	
Race:					0.028					0.014
Mexican American	200 (14.6%)	105 (15.2%)	44 (14.7%)	51 (13.4%)		244 (15.5%)	140 (17.2%)	49 (14.3%)	55 (13.3%)	
Other Hispanic	156 (11.4%)	70 (10.1%)	34 (11.3%)	52 (13.7%)		182 (11.6%)	84 (10.3%)	43 (12.6%)	55 (13.3%)	
Non-Hispanic White	527 (38.4%)	262 (37.9%)	123 (41.0%)	142 (37.4%)		575 (36.6%)	287 (35.2%)	127 (37.1%)	161 (39.0%)	
Non-Hispanic Black	273 (19.9%)	126 (18.2%)	55 (18.3%)	92 (24.2%)		343 (21.8%)	164 (20.1%)	87 (25.4%)	92 (22.3%)	
Other race	215 (15.7%)	128 (18.5%)	44 (14.7%)	43 (11.3%)		226 (14.4%)	140 (17.2%)	36 (10.5%)	50 (12.1%)	
Marital state:					<0.001					0.512
Married/living with a partner	925 (67.5%)	416 (60.2%)	226 (75.3%)	283 (74.5%)		933 (59.4%)	474 (58.2%)	211 (61.7%)	248 (60.0%)	
Widowed/divorced/ Separated/never married/NA	446 (32.5%)	275 (39.8%)	74 (24.7%)	97 (25.5%)		637 (40.6%)	341 (41.8%)	131 (38.3%)	165 (40.0%)	
Estradiol level	25.3 [20.3;31.6]	25.3 [20.4;31.3]	25.4 [20.5;31.4]	25.4 [19.8;32.2]	0.982	31.0 [7.14;95.7]	64.6 [32.1;131]	18.2 [6.24;94.8]	6.27 [3.30;10.9]	<0.001
DII	0.72 [−0.55;1.97]	0.75 [−0.47;2.04]	0.61 [−0.59;1.88]	0.69 [−0.70;1.88]	0.266	1.56 [0.38;2.57]	1.56 [0.42;2.58]	1.55 [0.38;2.46]	1.58 [0.23;2.63]	0.796
Uric acid level	6.00 [5.20;6.90]	6.00 [5.35;6.80]	5.90 [5.00;6.80]	6.00 [5.10;7.00]	0.340	4.70 [4.00;5.40]	4.50 [3.90;5.20]	4.70 [4.00;5.57]	4.90 [4.10;5.90]	<0.001
HUA (yes)	275 (20.1%)	137 (19.8%)	55 (18.3%)	83 (21.8%)	0.513	211 (13.4%)	71 (8.71%)	49 (14.3%)	91 (22.0%)	<0.001
UAT (yes)	841 (61.3%)	429 (62.1%)	180 (60.0%)	232 (61.1%)	0.818	519 (33.1%)	228 (28.0%)	116 (33.9%)	175 (42.4%)	<0.001
BMI	28.0 [24.4;32.0]	27.9 [24.0;32.0]	28.3 [25.4;31.4]	27.8 [24.7;32.8]	0.283	28.9 [23.9;34.8]	28.2 [23.1;34.0]	29.5 [24.8;35.8]	29.9 [25.1;36.0]	<0.001
TC level	189 [160;216]	186 [160;214]	197 [170;225]	187 [153;213]	<0.001	187 [164;213]	176 [154;198]	201 [178;230]	204 [179;226]	<0.001
TG level	100 [65.0;155]	96.0 [60.0;152]	103 [70.8;166]	105 [68.8;154]	0.051	83.0 [56.0;123]	71.0 [49.0;107]	93.5 [61.0;133]	102 [72.0;155]	<0.001
TyG level	9.18 [9.01;9.38]	9.13 [8.95;9.31]	9.26 [9.08;9.46]	9.25 [9.07;9.42]	<0.001	9.13 [8.96;9.31]	9.02 [8.88;9.17]	9.23 [9.06;9.40]	9.26 [9.12;9.44]	<0.001
eGFR level	901 [628;1398]	958 [664;1521]	882 [627;1274]	848 [561;1268]	<0.001	910 [572;1465]	881 [545;1470]	934 [627;1486]	937 [588;1448]	0.405
Alcohol status (yes)	210 (15.3%)	93 (13.5%)	55 (18.3%)	62 (16.3%)	0.120	93 (5.92%)	46 (5.64%)	27 (7.89%)	20 (4.84%)	0.186
Smoking status:					<0.001					<0.001
Never	668 (48.7%)	389 (56.3%)	131 (43.7%)	148 (38.9%)		1023 (65.2%)	585 (71.8%)	199 (58.2%)	239 (57.9%)	
Former	357 (26.0%)	127 (18.4%)	85 (28.3%)	145 (38.2%)		249 (15.9%)	89 (10.9%)	62 (18.1%)	98 (23.7%)	
Current	346 (25.2%)	175 (25.3%)	84 (28.0%)	87 (22.9%)		298 (19.0%)	141 (17.3%)	81 (23.7%)	76 (18.4%)	
Physical activity:					0.041					0.004
Inactive	670 (48.9%)	320 (46.3%)	136 (45.3%)	214 (56.3%)		970 (61.8%)	484 (59.4%)	213 (62.3%)	273 (66.1%)	
Moderate	287 (20.9%)	148 (21.4%)	68 (22.7%)	71 (18.7%)		353 (22.5%)	196 (24.0%)	72 (21.1%)	85 (20.6%)	
Vigorous	90 (6.56%)	45 (6.51%)	24 (8.00%)	21 (5.53%)		47 (2.99%)	20 (2.45%)	20 (5.85%)	7 (1.69%)	
Both moderate and vigorous	324 (23.6%)	178 (25.8%)	72 (24.0%)	74 (19.5%)		200 (12.7%)	115 (14.1%)	37 (10.8%)	48 (11.6%)	
Hypertension (yes)	506 (36.9%)	151 (21.9%)	120 (40.0%)	235 (61.8%)	<0.001	545 (34.7%)	151 (18.5%)	146 (42.7%)	248 (60.0%)	<0.001
Diabetes (yes)	229 (16.7%)	39 (5.64%)	64 (21.3%)	126 (33.2%)	<0.001	223 (14.2%)	53 (6.50%)	56 (16.4%)	114 (27.6%)	<0.001
Liver (yes)	69 (5.03%)	18 (2.60%)	15 (5.00%)	36 (9.47%)	<0.001	65 (4.14%)	11 (1.35%)	20 (5.85%)	34 (8.23%)	<0.001
Cancer (yes)	68 (4.96%)	6 (0.87%)	13 (4.33%)	49 (12.9%)	<0.001	91 (5.80%)	21 (2.58%)	21 (6.14%)	49 (11.9%)	<0.001
CKD (yes)	109 (7.95%)	37 (5.35%)	22 (7.33%)	50 (13.2%)	<0.001	169 (10.8%)	76 (9.33%)	36 (10.5%)	57 (13.8%)	0.057

Among the female participants, there were significant differences in E2, TC, TG, TyG, and HUA among the 20–45-year, 45–55-year, and 55–65-year groups (p<0.001). Compared with women aged 20–45 years, women aged 45–55 years and 55–65 years had lower E2 levels; higher TC, TG, and TyG levels; and a greater incidence of HUA. However, there were no significant differences in E2, TG, or HUA among the male participants in the 20–45-year-, 45–55-year, and 55–65-year-old age groups.

### Correlations of E2 with HUA and TG, TC, and TyG levels

Multivariate logistic regression analysis of E2 and HUA incidence was performed for the female population. For comparison, these analyses were similarly carried out in the male group and total population. Compared with the male and female subgroups, the total population model included an additional covariate, gender, to control for potential effects. After all potential confounders, in the female subgroup, ln(E2) was significantly associated with HUA in both weighted (OR=0.82, *p*=0.035) and unweighted models (OR=0.85, *p*=0.012). In the total population, ln(E2) was significantly associated with HUA both in weighted (OR=0.81, *p*=0.012) and unweighted models (OR=0.82, *p*<0.001), but not in the male group (weighted: OR=1.04, *p*=0.9; unweighted: OR=0.97, *p*=0.9) (**Table 2**).

**Table 2:** Associations of E2 with HUA.

Characteristic	Model1			Model2			Model3		
	OR	95% CI	*p*-value	OR	95% CI	*p*-value	OR	95% CI	*p*-value
Unweighted									
Total	0.89	0.82, 0.97	0.006*	0.82	0.73, 0.91	<0.001*	0.82	0.74, 0.91	<0.001*
Male	1.39	0.97, 2.03	0.080	0.98	0.67, 1.44	>0.9	0.97	0.66, 1.43	0.9
Female	0.85	0.77, 0.93	<0.001*	0.84	0.74, 0.96	0.010*	0.85	0.75, 0.96	0.012*
Weighted									
Total	0.91	0.83, 0.99	0.033*	0.81	0.71, 0.92	0.004*	0.81	0.69, 0.94	0.012*
Male	1.65	0.93, 2.93	0.086	1.06	0.56, 2.02	0.8	1.04	0.50, 2.20	0.9
Female	0.86	0.78, 0.95	0.004*	0.82	0.69, 0.96	0.021*	0.82	0.68, 0.98	0.035*

*p<0.05. Model 1: crude model. Model 2: adjusted for age, BMI, race, PIR, education level, marital status, alcohol status, smoking status, and physical activity (for total popular gender was added). Model 3: adjusted for age, BMI, race, PIR, education level, marital status, alcohol status, smoking status, physical activity, cancer, CKD, diabetes, hypertension, and liver disease (for total popular gender was added).

As no correlation between E2 and HUA incidence was detected in the male subgroup, regression analyses of ln(E2) and mediator variables were only performed for the female subgroup and total population. After adjusting for all potential confounders, in the female subgroup, E2 was significantly associated with TC (weighted: Beta=−2.2, *p*=0.032; unweighted: Beta=−2.0, *p*=0.006), TG (weighted: Beta=−4.8, *p*=0.019; unweighted: Beta=−5.4, *p*=0.025), and TyG (weighted: Beta=−0.01, *p*=0.048; unweighted: Beta=−0.01, *p*=0.009) in both weighted and unweighted models, and the results were similar in the total population (TC, weighted: Beta=−3.7, *p*<0.001; unweighted: Beta=−3.9, *p*<0.001). TG, weighted: Beta=−6.2, *p*=0.001; unweighted: Beta=−6.8, *p*=0.002. TyG, weighted: Beta=−0.02, *p*=0.002; unweighted: Beta=−0.02, *p*<0.001) (**Table 3**).

**Table 3:** Associations of E2 with TG, TC, and TyG.

Characteristic	Model1			Model2			Model3		
	Beta	95% CI	*p*-value	Beta	95% CI	*p*-value	Beta	95% CI	*p*-value
TG (unweighted)									
Total	−8.1	−12, −4.0	<0.001*	−7.1	−11, −2.8	0.001*	−6.8	−11, −2.5	0.002*
Female	−8.1	−12, −4.1	<0.001*	−6.3	−11, −1.5	0.010*	−5.4	−10, −0.67	0.025*
TG (weighted)									
Total	−6.8	−10, −3.6	<0.001*	−6.8	−10, −3.5	<0.001*	−6.2	−9.4, −3.0	0.001*
Female	−7.2	−10, −4.4	<0.001*	−6.1	−10, −1.8	0.008*	−4.8	−8.8, −0.90	0.019*
TC (unweighted)									
Total	−6.0	−7.2, −4.7	<0.001*	−3.9	−5.2, −2.5	<0.001*	−3.9	−5.3, −2.6	<0.001*
Female	−6.0	−7.3, −4.8	<0.001*	−2.0	−3.5, −0.57	0.006*	−2.0	−3.5, −0.57	0.006*
TC (weighted)									
Total	−5.6	−7.1, −4.2	<0.001*	−3.6	−5.1, −2.1	<0.001*	−3.7	−5.3, −2.1	<0.001*
Female	−5.8	−7.2, −4.4	<0.001*	−2.2	−4.1, −0.31	0.025*	−2.2	−4.1, −0.21	0.032*
TyG (unweighted)									
Total	−0.05	−0.06, −0.04	<0.001*	−0.03	−0.04, −0.02	<0.001*	−0.02	−0.03, −0.02	<0.001*
Female	−0.06	−0.07, −0.05	<0.001*	−0.02	−0.03, −0.01	0.001*	−0.01	−0.02, 0.00	0.009*
TyG (weighted)									
Total	−0.05	−0.06, −0.04	<0.001*	−0.03	−0.04, −0.01	0.001*	−0.02	−0.03, −0.01	0.002*
Female	−0.05	−0.06, −0.04	<0.001*	−0.02	−0.04, −0.01	0.009*	−0.01	−0.03, 0.00	0.048*

*p<0.05. Model 1: crude model. Model 2: adjusted for age, BMI, race, PIR, education level, marital status, alcohol status, smoking status, and physical activity (for total popular gender was added). Model 3: adjusted for age, BMI, race, PIR, education level, marital status, alcohol status, smoking status, physical activity, cancer, CKD, diabetes, hypertension, and liver disease (for total popular gender was added).

### Associations between E2 and HUA

The trend in the E2 quintile with HUA incidence was evaluated simultaneously in the female group and total population. In the female subgroup, the incidence of HUA tended to increase and then decrease when ln(E2) decreased from Q4 to Q1 (*p* for trend=0.003). In addition, in the total population, the incidence of HUA tended to decrease first, increase, and then decrease when ln(E2) decreased from Q4 to Q1(*p* for trend=0.018). After controlling for all covariates, the incidence of HUA tended to increase when ln(E2) decreased from Q4 to Q1 in the female subgroup (*p* for trend=0.026), and the trend was broadly similar in the total population (*p* for trend=0.009) (**Table 4**).

**Table 4:** Associations of the E2 quintiles with HUA incidence.

Characteristic	No. of cases	Model1			Model2			Model3		
		OR	95% CI	*p*-value	OR	95% CI	*p*-value	OR	95% CI	*p*-value
Total										
Q1	593	1.91	1.21, 3.02	0.007*	2.45	1.51, 3.97	0.003*	2.44	1.22, 4.91	0.027*
Q2	553	2.27	1.53, 3.35	<0.001*	1.56	0.78, 3.12	0.2	1.55	0.60, 4.01	0.2
Q3	586	1.84	1.26, 2.69	0.003*	1.18	0.65, 2.14	0.5	1.18	0.52, 2.68	0.6
Q4	603	2.22	1.41, 3.50	0.001*	1.30	0.71, 2.36	0.3	1.25	0.53, 2.95	0.5
Q5	606		Ref			Ref			Ref	
P for trend			0.018*			0.003*			0.009*	
Female										
Q1	305	1.69	0.86, 3.30	0.12	2.55	1.02, 6.38	0.046*	2.64	0.90, 7.73	0.067*
Q2	316	3.06	1.50, 6.25	0.003*	2.34	1.00, 5.51	0.051*	2.28	0.80, 6.49	0.095*
Q3	319	2.19	1.15, 4.18	0.019*	1.71	0.76, 3.87	0.2	1.73	0.67, 4.49	0.2
Q4	303	1.15	0.58, 2.30	0.7	1.23	0.53, 2.85	0.6	1.26	0.48, 3.29	0.5
Q5	327		Ref			Ref			Ref	
P for trend			0.003*			0.016*			0.026*	

*p<0.05. Model 1: crude model. Model 2: adjusted for age, BMI, race, PIR, education level, marital status, alcohol status, smoking status, and physical activity (for total popular gender was added). Model 3: adjusted for age, BMI, race, PIR, education level, marital status, alcohol status, smoking status, physical activity, cancer, CKD, diabetes, hypertension, and liver disease (for total popular gender was added).

**Figure 2:**
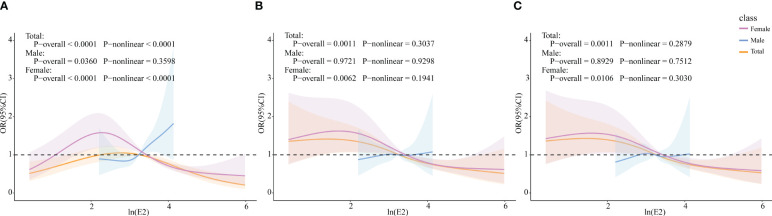
Associations of E2 with HUA via RCS analysis. **(A)** Model 1, crude model. **(B)** Model 2, adjusted for age, BMI, race, PIR, education level, marital status, alcohol status, smoking status, and physical activity (for total popular gender was added). **(C)** Model 3, adjusted for age, BMI, race, PIR, education level, marital status, alcohol status, smoking status, physical activity, cancer, CKD, diabetes, hypertension, and liver disease (for total popular gender was added).

The RCS model was used to further explore whether there was a non-linear association between E2 and HUA incidence. In the female group and total population, ln(E2) exhibited a non-linear relationship with the incidence of HUA (*p*-overall<0.0001, *p*-non-linear<0.0001), whereas it was linearly associated with HUA (*p*-overall=0.0360, *p*-non-linear=0.3598) in the male group. After controlling for all the covariates, ln(E2) was shown to be linearly correlated with the incidence of HUA in the female subgroup (*p*-overall=0.0106, *p*-non-linear=0.3030) and total population (*p*-overall=0.0011, *p*-non-linear=0.2879). However, the difference in expression in the male subgroup was not significant (*p*-overall=0.8929, *p*-non-linear=0.7512), which was consistent with the results of multivariate logistic regression (**Figure 2**). Similarly, in the female group, ln(E2)=1.77 (E2 = 5.87 pg/mL) was regarded as the cutoff point. This meant that when E2 was lower than 5.87 pg/mL, the risk of HUA was consistent with the trend of E2. When E2 was greater than 5.87 pg/mL, the risk of HUA was opposite to the trend of E2.

### Associations between TG, TC, TyG, and HUA incidence

RCS models were constructed to explore whether there were non-linear associations between TG, TC and TyG, and HUA. In the female subgroup, TC was linearly correlated with HUA (*p*-overall=0.0019, *p*-non-linear=0.6075), whereas TG and TyG were non-linearly correlated with HUA (TG: *p*-overall<0.0001, *p*-non-linear<0.0001; TyG: *p*-overall<0.0001, *p*-non-linear=0.0160). The results were similar in the total population (TC: *p*-overall<0.0001, *p*-non-linear=0.7917; TG: *p*-overall<0.0001, *p*-non-linear<0.0001; TyG: *p*-overall<0.0001, *p*-non-linear<0.0001).

**Figure 3:**
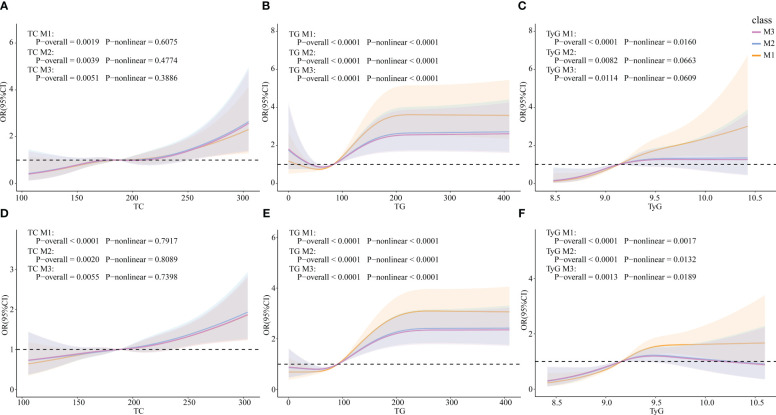
Associations of TG, TC, and TyG with HUA via RCS analysis. **(A)** TC in females. **(B)** TG in females. **(C)** TyG in females. **(D)** TC in the total population. **(E)** TG in the total population. **(F)** TyG in the total population. M1: Crude model. M2: Adjusted for age, BMI, race, PIR, education level, marital status, alcohol status, smoking status, physical activity, cancer, CKD, diabetes, hypertension, and liver disease (for total popular gender was added). M3: Adjusted for ln(E2), age, BMI, race, PIR, education level, marital status, alcohol status, smoking status, physical activity, cancer, CKD, diabetes, hypertension, and liver disease (for total popular gender was added).

After controlling for all covariates (M2), TC and TyG were linearly correlated with HUA (TC: *p*-overall=0.0039, *p*-non-linear= 0.4774; TyG: *p*-overall=0.0082, *p*-non-linear=0.0663), whereas TG was non-linearly correlated with HUA (*p*-overall<0.0001, *p*-non-linear<0.0001) in the female group. In the total population, TC was linearly correlated with HUA (*p*-overall= 0.0020, *p*-non-linear=0.8089), whereas TG and TyG were non-linearly correlated with HUA (TG: *p*-overall<0.0001, *p*-non-linear<0.0001; TyG: *p*-overall<0.0001, *p*-non-linear=0.0132). After adding ln(E2) to the model 3 (M3), the results were similar to those of M2 in both the female group and the total population (**Figure 3**).

### Mediating effects of TG, TC, and TyG on E2 and HUA

Mediation analyses were performed to examine whether TC, TG, and TyG mediated the relationship between E2 and HUA. As the results showed, TC, TG, and TyG significantly mediated the relationship between ln(E2) and HUA in the female subgroup (TC, indirect effect: −0.00498, 19.9%, *p*=0.004; TG, indirect effect: −0.00291, 11.4%, *p*< 0.001; TyG, indirect effect: −0.00957, 38.6%, *p*< 0.001). After controlling for all the covariates, although the proportion of mediating effects decreased, the above mediating indicators were still statistically significant in the female subgroup (TC, indirect effect: −0.00148, 7.5%, *p*=0.008; TG, indirect effect: −0.00062, 3.1%, *p*=0.004; TyG, indirect effect: −0.00113, 5.6%, *p*=0.016). In the total population, TC and TG significantly mediated the relationship between ln(E2) and HUA (TC, indirect effect: −0.00476, 24.3%, *p*< 0.001; TG, indirect effect: −0.00238, 12.1%, *p*< 0.001). After controlling for all the covariates, the proportion of mediating effects decreased, but all three indicators showed a statistically significant mediating effect (TC, indirect effect: −0.00274, 9.9%, *p*< 0.001; TG, indirect effect: −0.00123, 4.4%, *p*< 0.001; TyG, indirect effect: −0.00174, 6.2%, *p*=0.012) (**Table 5**).

**Table 5:** TC, TG, and TyG levels mediate the association between E2 and HUA incidence.

		Total effect			Indirect effect			Direct effect			Proportion mediated (%)		
		Coefficient	95% CI	*p*-value	Coefficient	95% CI	*p*-value	Coefficient	95% CI	*p*-value	Coefficient	95% CI	*p*-value
TC	Total												
	Model 1*	-0.01963	-0.03406,-0.00725	< 0.001	-0.00476	-0.00735,-0.00240	< 0.001	-0.01486	-0.02967,-0.00218	0.020	24.3	11.6,63.7	< 0.001
	Model 2*	-0.02761	-0.04750,-0.01165	< 0.001	-0.00274	-0.00471,-0.00114	< 0.001	-0.02487	-0.04482,-0.00927	< 0.001	9.9	3.9,24.7	< 0.001
	Female												
	Model 1*	-0.02497	-0.04156,-0.01033	< 0.001	-0.00498	-0.00860,-0.00187	0.004	-0.01999	-0.03618,-0.00585	< 0.001	19.9	7.5,45.3	0.004
	Model 2*	-0.01986	-0.03874,-0.00577	0.004	-0.00148	-0.00332,-0.00025	0.008	-0.01837	-0.03662,-0.00432	0.008	7.5	1.3,22.9	0.012
TG	Total												
	Model 1*	-0.01967	-0.03480,-0.00720	< 0.001	-0.00238	-0.00599,-0.00123	< 0.001	-0.01730	-0.03102,-0.00385	0.008	12.1	5.0,51.7	< 0.001
	Model 2*	-0.02768	-0.04760,-0.01181	< 0.001	-0.00123	-0.00321,-0.00055	< 0.001	-0.02645	-0.04652,-0.01070	< 0.001	4.4	1.7,14.6	< 0.001
	Female												
	Model 1*	-0.02559	-0.04322,-0.00993	< 0.001	-0.00291	-0.00886,-0.00050	< 0.001	-0.02268	-0.03849,-0.00613	0.004	11.4	2.0,45.1	< 0.001
	Model 2*	-0.02009	-0.03875,-0.00611	0.004	-0.00062	-0.00414,-0.00018	0.004	-0.01946	-0.03724,-0.00475	0.004	3.1	0.8,28.4	0.008
TyG	Total												
	Model 1	-0.01985	-0.03462,-0.00724	< 0.001	-0.00762	-0.01058,-0.00532	< 0.001	-0.01223	-0.02686,0.00011	0.060	38.4	22.2,101.8	< 0.001
	Model 2*	-0.02803	-0.04826,-0.01163	< 0.001	-0.00174	-0.00337,-0.00048	0.012	-0.02630	-0.04635,-0.00966	< 0.001	6.2	1.9,16.6	0.012
	Female												
	Model 1*	-0.02480	-0.04181,-0.01022	< 0.001	-0.00957	-0.01354,-0.00563	< 0.001	-0.01523	-0.03080,-0.00131	0.028	38.6	21.3,87.6	< 0.001
	Model 2*	-0.02026	-0.03952,-0.00595	0.004	-0.00113	-0.00284,-0.00013	0.016	-0.01913	-0.03798,-0.00473	0.004	5.6	0.6,18.5	0.020

*p<0.05. Model 1: crude model. Model 2: adjusted for age, BMI, race, PIR, education level, marital status, alcohol status, smoking status, physical activity, cancer, CKD, diabetes, hypertension, and liver disease (for total popular gender was added).

### Associations between TG, TC, TyG, E2, and UAT

First, multivariate logistic regression analysis of TG, TC, TyG, and UAT was performed (**Supplementary Table 1**). In the overall population and the male and female subgroups, TC was positively correlated with UAT after all covariates were included, whether unweighted (total: OR=1.01, *p*<0.001; male: OR=1.01, *p*<0.001; female: OR=1.01, *p*<0.001) or weighted (total: OR=1.01, *p*=0.003; male: OR=1.01, *p*=0.030; female: OR=1.01, *p*=0.001). In the overall population and female subgroup, TyG was positively correlated with UAT after all covariates were included, whether unweighted (total: OR=1.91, *p*<0.001; female: OR=2.54, *p*<0.001) or weighted (total: OR=2.32, *p*=0.010; female: OR=3.97, *p*<0.001). For TG, after all covariates were included, unweighted TG and UAT showed a statistically significant positive correlation in the overall population and the male subgroup (total: OR=1.00, *p*=0.005; male: OR=1.00, *p=*0.002), and there was a statistically significant positive correlation between weighted TG and UAT in the male subgroup (male: OR=1.00, *p=*0.009). In general, there is a certain correlation between the three indicators related to glucolipid metabolism and UAT, the uric acid threshold that better reflects cardiovascular disease. TG had a stronger correlation with UAT in the male subgroup, and TyG had a stronger correlation with UAT in the female subgroup. There was no significant gender difference in the correlation between TC and UAT.

Subsequently, multivariate logistic regression analysis of ln(E2) and UAT was performed (**Supplementary Table 2**). With the inclusion of all covariates, E2 showed a statistically significant negative correlation with UAT in the overall population only in the unweighted model (OR=0.91, *p*=0.022). In contrast, E2 showed no correlation with UAT in the male and female subgroups or in the weighted condition.

**Figure 4:**
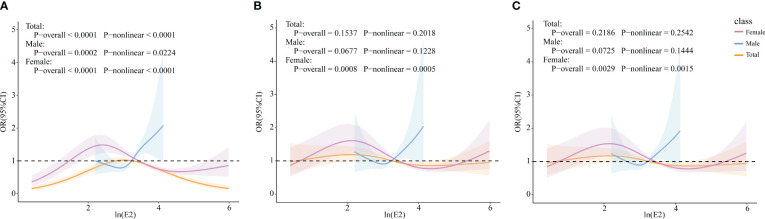
Associations of E2 with UAT via RCS analysis. **(A)** Model 1, Crude model. **(B)** Model 2, adjusted for age, BMI, race, PIR, education level, marital status, alcohol status, smoking status, and physical activity (for total popular gender was added). **(C)** Model 3, adjusted for age, BMI, race, PIR, education level, marital status, alcohol status, smoking status, physical activity, cancer, CKD, diabetes, hypertension, and liver disease (for total popular gender was added).

RCS models were constructed to explore whether there were non-linear associations between ln(E2) and UAT. According to the results in **Figure 4**, after all covariates were included, E2 and UAT in the female population were non-linearly and significantly correlated (*p*-overall=0.0029, *p*-non-linear=0.0015). There are two inflection points in the curve. When ln(E2)<2.167 or >4.474, the trend of UAT is consistent with the change in E2. When 2.167 < ln(E2) < 4.474, the trend of UAT is opposite to the E2 change.

Based on the RCS results, the female population was categorized into ln(E2)<2.167 (n=461) and ln(E2)>2.167 (n=1109) groups. Multivariate logistic regression analysis of ln(E2) and UAT was rerun for these two components, respectively. The results in **Table 6** showed that among women with ln(E2)<2.167, E2 was positively correlated with UAT after all covariates were included, whether unweighted (OR=1.55, *p*=0.018) or weighted (OR=1.54, *p*=0.032). In the female population with ln(E2)>2.167, unweighted E2 was negatively correlated with UAT (OR=0.84, *p*=0.022). Unfortunately, there was no statistically significant correlation between weighted E2 and UAT.

**Table 6:** Associations of E2 with UAT in the female group.

**Characteristic**	**Model1**			**Model2**			**Model3**		
	**OR**	**95% CI**	***p*-value**	**OR**	**95% CI**	***p*-value**	**OR**	**95% CI**	***p*-value**
Unweighted									
ln(E2) < 2.167	1.83	1.34, 2.53	<0.001*	1.56	1.10, 2.24	0.015*	1.55	1.08, 2.23	0.018*
ln(E2) > 2.167	0.69	0.61, 0.79	<0.001*	0.82	0.71, 0.95	0.010*	0.84	0.72, 0.97	0.022*
Weighted									
ln(E2) < 2.167	2.03	1.44, 2.87	<0.001*	1.61	1.09, 2.40	0.022*	1.54	1.05, 2.26	0.032*
ln(E2) > 2.167	0.74	0.62, 0.88	0.001*	0.86	0.71, 1.05	0.130	0.90	0.72, 1.13	0.300

*p<0.05. Model 1: crude model. Model 2: adjusted for age, BMI, race, PIR, education level, marital status, alcohol status, smoking status, and physical activity (for total popular gender was added). Model 3: adjusted for age, BMI, race, PIR, education level, marital status, alcohol status, smoking status, physical activity, cancer, CKD, diabetes, hypertension, and liver disease (for total popular gender was added).

Finally, mediation analyses were performed to examine whether TC, TG, and TyG mediated the relationship between E2 and UAT in both the ln(E2)<2.167 and ln(E2)>2.167 female populations. The results of **Table 7** show that after including all covariates, only TyG mediated the correlation between E2 and UAT in the population of women with ln(E2) > 2.167 (indirect effect: −0.00343, 9.3%, *p*= 0.048).

**Table 7:** TC, TG, and TyG levels mediate the association between E2 and UAT in the female group.

		Total effect			Indirect effect			Direct effect			Proportion mediated (%)		
		Coefficient	95% CI	*p*-value	Coefficient	95% CI	*p*-value	Coefficient	95% CI	*p*-value	Coefficient	95% CI	*p*-value
TC	ln(E2) < 2.167												
	Model 1*	0.11074	0.06344,0.15116	<0.001	0.00668	0.00020,0.01623	0.044	0.10407	0.05741,0.14419	<0.001	6.0	0.2,18.0	0.044
	Model 2	0.07052	0.00994,0.11259	0.020	0.01303	0.00228,0.02718	0.004	0.05749	-0.00429,0.10919	0.064	18.5	2.9,92.7	0.024
	ln(E2) > 2.167												
	Model 1*	-0.08264	-0.09979,-0.05586	<0.001	-0.00902	-0.01456,-0.00424	<0.001	-0.07362	-0.09295,-0.04715	<0.001	10.9	5.0,21.0	<0.001
	Model 2	-0.03666	-0.06914,-0.00681	0.012	-0.00245	-0.00660,0.00149	0.208	-0.03421	-0.06644,-0.00449	0.012	6.7	-5.3,39.2	0.212
TG	ln(E2) < 2.167												
	Model 1	0.11149	0.06386,0.15152	<0.001	0.00407	-0.00769,0.01686	0.488	0.10742	0.05811,0.14903	<0.001	3.6	-7.3,16.9	0.488
	Model 2	0.07179	0.01251, 0.12484	0.020	0.00155	-0.01137,0.01172	0.748	0.07025	0.01207,0.12305	0.024	2.2	-28.8,27.0	0.752
	ln(E2) > 2.167												
	Model 1	-0.08418	-0.09800,-0.05795	<0.001	-0.00572	-0.01673,0.00017	0.056	-0.07846	-0.09502,-0.05086	<0.001	6.8	-0.2,21.8	0.058
	Model 2	-0.03677	-0.06969,-0.00673	0.016	-0.00052	-0.00527,0.00097	0.472	-0.03625	-0.06954,-0.0606	0.012	1.4	4.0,19.3	0.480
TyG	ln(E2) < 2.167												
	Model 1*	0.11093	0.06358,0.15111	<0.001	0.00734	0.00059,0.01891	0.024	0.10359	0.05290,0.14368	<0.001	6.6	0.5,21	0.024
	Model 2	0.07110	0.01189,0.12227	0.020	0.00775	-0.00047,0.02180	0.076	0.06335	0.00241,0.11472	0.048	10.9	-3.5,56.4	0.096
	ln(E2) > 2.167												
	Model 1*	-0.08199	-0.09944,-0.5532	<0.001	-0.02347	-0.03134,-0.01589	<0.001	-0.05852	-0.08039,-0.3114	<0.001	28.6	17.5,48.3	<0.001
	Model 2*	-0.03697	-0.06949,-0.00718	0.012	-0.00343	-0.00822,-0.00030	0.036	-0.03354	-0.06568,-0.00382	0.016	9.3	0.3,39.1	0.048

*p<0.05. Model 1: crude model. Model 2: adjusted for age, BMI, race, PIR, education level, marital status, alcohol status, smoking status, physical activity, cancer, CKD, diabetes, hypertension, and liver disease (for total popular gender was added).

## Discussion

This study used the NHANES public database 2013–2016 to investigate the relationship between E2 and the incidence of HUA in adults aged 25–65 years in the U.S., especially in adult women, and to explore whether this relationship is partially mediated by TC, TG, and TyG.

In this study, the correlations between E2 and the incidence of HUA and the mediating effects of TC, TG, and TyG were analyzed via various methods. Descriptive statistics showed that for people aged 25–65 years, the prevalence of HUA was 20.1% in the male population and 13.4% in the female population. This finding is similar to the results of previous investigations (6–8). In the female group, 45- to 55-year-old perimenopausal and 55- to 65-year-old postmenopausal women had lower E2 levels and higher HUA incidence than 25- to 45-year-old women. However, in the male group, no statistically significant differences in E2 level or HUA incidence were found among the age groups.

Previous studies have shown that the prevalence of HUA in middle-aged women has increased dramatically from the late menopausal transition stage (10), and the serum uric acid level in postmenopausal women was 0.34 mg/dL higher than that in premenopausal women (42). A decrease in E2 during perimenopause and postmenopause is considered to be the main cause of this phenomenon. A cross-sectional study showed that E2 levels were independently associated with the prevalence of HUA in female participants (12). In this study, the results of multivariate logistic regression analysis and the RCS model also supported this view. In the female subgroup, the incidence of HUA was negatively correlated with E2 levels and showed a linear correlation. This correlation was not detected in the male group. In addition, in the total population, E2 was negatively correlated with the incidence of HUA, and after adjusting for all confounding factors (including gender), its negative effect was more prominent and statistically significant. This finding proved that a change in the E2 level had an important impact on the incidence of HUA, which was especially obvious in the female group. In addition, the RCS model showed that for women, an E2 greater than 5.87 pg/mL was a protective factor against HUA, and a level lower than 5.87 pg/mL was a risk factor. A Chinese longitudinal cohort study showed that E2 levels in women began to decline 8 years before menopause, sharply declined 2 years before menopause, and stabilized at a low level 2 years after menopause. In addition, a study showed that the average estradiol level of premenopausal women was approximately 108.57 pg/mL, decreased to 84.28 pg/mL in the perimenopausal period, approximately 41.38 pg/mL within 1 year after menopause, and stabilized at 11.91 pg/mL after 2–8 years of menopause (43). In our study, the median level of E2 in postmenopausal women was 6.27 pg/mL. Combined with the RCS results, our cutoff value was 5.87 pg/mL, which was lower than the level of 2–8 years after menopause and lower than the median of postmenopausal patients. This suggests that for perimenopausal women and at least some postmenopausal women, the change in E2 is negatively correlated with the prevalence of HUA.

The most likely biological mechanism by which sex hormones cause changes in UA levels is their effects on renal tubular and intestinal processing of uric acid. An early study showed that estradiol inhibited the urate reabsorptive transporters urate transporter 1 (URAT1) and glucose transporter 9 (GLUT9) protein expression in mouse kidneys. Progesterone was able to reduce sodium-coupled monocarboxylate transporter 1 (SMCT1) protein expression. Both of them reduce SUA levels by decreasing the UA reabsorption capacity of the kidney (44). A previous BioCycle study showed an inverse relationship between progesterone and UA levels (45). A recent study by Lei Liu et al. (46) showed that in addition to regulating renal uric acid processing, estradiol also promotes intestinal ATP-binding cassette subfamily G member 2 (ABCG2) expression, thereby increasing uric acid excretion. A study by Changqian Liu et al. (47) showed that E2 played a crucial protective role in HUA induced by polychlorinated biphenyl 138 in female mice. Changes in sex hormones in the perimenopausal period are characterized by increases in follicle-stimulating hormone (FSH) levels, followed by decreases in E2 and progesterone levels. Based on the above studies, changes in E2 levels may be the main sex hormone causes of perimenopausal and postmenopausal hyperuricemia. However, there is also an early study showing that E2 treatment fails to play a role in renal UA metabolism (48). This may be related to our finding that there is a dose turning point in the effect of E2 on HUA (5.87 pg/mL in this study). The potential biological regulation mechanism of sex hormones on the UA level is still unclear. More research results are still needed for interpretation.

Based on previous studies, a reduction in E2 could affect women’s lipid metabolism function, increasing the prevalence of cardiovascular disease (14–16). Similarly, studies have shown that glucolipid metabolism disorders have a close impact on uric acid levels. A retrospective cohort study in China showed a positive correlation between SUA levels and TC (49). Statins, drugs with cholesterol-lowering effects, were confirmed to be significantly associated with a reduction in SUA levels (17). A 5-year cohort study revealed that there was a temporal relationship between abnormal TG levels and HUA. A change in TG precedes a change in SUA, which means that TG can unilaterally affect SUA (18). TyG is a well-established biological indicator for evaluating insulin resistance and cardiovascular risk (50). In non-obese patients with type II diabetes, TyG was confirmed to be positively correlated with SUA levels and may be more predictive of HUA in non-obese patients with type II diabetes than the homeostasis model assessment of insulin resistance (HOMA-IR) (19). In patients with HUA, the TyG score was also found to mediate the increase in SUA levels (51). Some studies have also shown that changes in UA can also affect lipid and glucose metabolism (52). SUA levels can predict the development of LDL-C and TG levels (53) and are recognized as strong and independent risk factors for diabetes (54, 55). In summary, there is an interactive and complex relationship between UA and glucolipid metabolism.

The potential biological mechanism of the interaction between UA, lipids, and glucose has been studied. On the one hand, high levels of lipids lead to more free fatty acids and promote synthesis, which is related to *de novo* purine synthesis, thereby accelerating the production of UA (56). In addition, dyslipidemia may lead to excessive activity of xanthine oxidoreductase and promote uric acid production (57–59). High levels of glucose can cause the proximal renal tubules to increase the reabsorption of glucose, uric acid, chloride, etc., which may be one of the mechanisms for the increase in SUA (60). By inhibiting sodium-glucose cotransporter-2 (SGLT-2) to weaken the proximal reabsorption of sodium and glucose, UA excretion can be promoted (61). On the other hand, during the conversion of hypoxanthine to SUA determined by xanthine oxidase, the increase of superoxide anion causes oxidative stress, leading to mitochondrial dysfunction and citrate release, promoting lipid synthesis (62). In addition, excessive activation of lipid metabolism-related pathways, such as C–X–C motif chemokine ligand 13 (CXCL13) and sterol regulatory element-binding protein 1C (SREBP-1c), and endoplasmic reticulum stress may also be involved in high UA-induced fat accumulation (63–65). For glucose metabolism, many studies have provided evidence that HUA can cause oxidative damage and inhibit insulin secretion in pancreatic β cells (66, 67). However, whether there is a time relationship and causal relationship between the changes of UA and glucolipid metabolism during changes in estrogen in perimenopausal and postmenopausal women needs further study to clarify.

The results of this study showed that E2 was negatively correlated with TC, TG, and TyG in the female population. After controlling for all covariates, the results were still statistically significant. The RCS model results showed that in the female subgroup, TG and HUA exhibited a non-linear correlation, whereas TC and TyG were linearly correlated with HUA after controlling for all potential influencing factors. Combined with the mediation analysis results, TG, TC, and TyG played partial mediating roles in the influence of E2 on the incidence of HUA. After controlling for all covariates, the mediating effects were 3.1%, 7.5%, and 5.6% in the female group and 4.4%, 9.9%, and 6.2% in the total population. In summary, our results suggest that changes in E2 levels in women are closely related to glucolipid metabolism and UA levels. Therefore, in clinical practice, certain interventions, such as supplementing a certain dose of E2, to increase UA levels, ameliorate glucolipid metabolism disorders, and prevent possible cardiovascular or metabolic diseases in perimenopausal and postmenopausal women, should be implemented in a timely manner or even in advance. In addition, women have large changes in sex hormones at different physiological stages, such as women in pregnancy who were not included in this study. Fluctuations in uric acid levels were also observed in this state (68). The causality and potential mechanisms need further study.

In the exploration of the UAT, the uric acid threshold that is more closely associated with cardiovascular disease, we found that TG had a stronger correlation with UAT in the male subgroup, and TyG had a stronger correlation with UAT in the female subgroup. There was no significant sex difference in the correlation between TC and UAT. In the female population, there was a non-linear relationship between E2 and UAT. When ln(E2)<2.167 (E2<8.73 pg/mL), E2 was positively correlated with UAT. When ln(E2)>2.167 (E2>8.73 pg/mL), E2 was negatively correlated with UAT. The correlation between E2 and UAT is more difficult to detect than the clinical HUA diagnostic criteria and appears to be significantly non-linear in the female population. This may be due to the fact that changes in uric acid levels associated with estrogenic changes in the female population are not directly linked to cardiovascular disease. Therefore, the specificity of UAT as a threshold for predicting cardiovascular risk may be responsible for the attenuated correlation with E2 changes. Furthermore, in the female population, TC, TG, and TyG were all shown to be involved in the correlation between E2 and HUA, whereas only TyG was found to have a mediating effect on E2 and UAT. However, TyG exerted a 9.3% mediating effect in the correlation between E2 and UAT, which was greater than the mediating effect in the correlation between E2 and HUA (5.6%). This is inseparable from the ability of TyG itself to evaluate cardiovascular risk. At the same time, this finding also suggests that TyG may be a good breakthrough point in future research on the role of female E2 changes on metabolic diseases such as cardiovascular disease or HUA.

The data in this study came from an extensive sample sampling survey, and the influence of confounding factors was adjusted twice during the statistical analysis; therefore, the results were reliable. This study has certain limitations. First, due to the cross-sectional nature of this study, it was limited to establishing the temporal and causal relationship between changes in E2 levels, glucolipid metabolism, and HUA in women. Further longitudinal cohort studies are needed to assess changes in glucolipid metabolism and HUA before and after menopause. Then, we cannot clarify the specific pathway by which E2 affects the incidence of HUA or the specific mechanism involved in the mediating effect of TC, TG, and TyG. Further in-depth research is needed. In fact, the use of diuretics has an impact on UA levels (69). Unfortunately, NHANES does not provide this part of the data, which is a strong limitation of this study. In addition, in multivariate analysis, although most potential confounders were adjusted for, it cannot be excluded that there are still other potential factors that may have an impact on the results.

## Conclusions

The results of this study showed that female E2 levels were negatively correlated with the incidence of HUA. Low E2 levels during perimenopause and postmenopause were associated with the risk of HUA. In addition, we found that TC, TG, and TyG, which are markers of glucolipid metabolism, played a mediating role in the association between E2 and HUA. Our findings highlight the important impact of changes in female E2 levels on health levels, especially metabolism, and the interaction between glucose, lipids, and uric acid levels. Due to the limitations of the cross-sectional nature of this study, it is necessary to further clarify the temporal and causal relationships between E2, glucolipid metabolism, and UA levels in women in future studies. Whether this class of HUA due to changes in sex hormones has a different biological mechanism or characteristics from conventional HUA deserves to be investigated. It is also necessary to actively explore appropriate prevention indicators and prevention strategies for women in the perimenopausal and even premenopausal periods. In addition, for clinical practice, we should pay full attention to the complex effects that may exist between perimenopausal and postmenopausal, HUA, and cardiovascular disease. For women with possible morbidity trends, such as abnormal cardiovascular or metabolic-related indicators, appropriate prevention strategies can be implemented in the premenopausal period to reduce the occurrence of the disease and its related consequences.

## Data availability statement

The original contributions presented in the study are included in the article/**Supplementary Material**. Further inquiries can be directed to the corresponding author.

## Author contributions

CZ: Conceptualization, Data curation, Methodology, Writing – original draft. HQ: Conceptualization, Data curation, Formal analysis, Writing – original draft. YWC: Data curation, Formal analysis, Writing – original draft. XL: Validation, Writing – original draft. YLC: Visualization, Writing – original draft. LG: Supervision, Writing – review & editing.
