# Supplementary material for: Associations between estradiol and hyperuricemia and the mediating effects of TC, TG, and TyG: NHANES 2013–2016
Source: Front Endocrinol (Lausanne). 2024 Aug 7;15:1422470. doi: 10.3389/fendo.2024.1422470 (PMC11335549; doi:10.3389/fendo.2024.1422470)
Supplement: Supplementary file 1 [file Table_1.docx]

**Supplementary Table 1** Associations of TC, TG, and TyG with UAT

| **Characteristic** | |  | **Model1** | | | |  | **Model2** | | |  | **Model3** | | |
| --- | --- | --- | --- | --- | --- | --- | --- | --- | --- | --- | --- | --- | --- | --- |
|  |  |  | **OR** | | **95% CI** | **p-value** |  | **OR** | **95% CI** | **p-value** |  | **OR** | **95% CI** | **p-value** |
| unweighted |  |  |  | |  |  |  |  |  |  |  |  |  |  |
| TC | Total |  | 1.01 | | 1.00, 1.01 | <0.001* |  | 1.01 | 1.00, 1.01 | <0.001* |  | 1.01 | 1.00, 1.01 | <0.001* |
|  | Male |  | 1.01 | | 1.00, 1.01 | <0.001* |  | 1.01 | 1.00, 1.01 | <0.001* |  | 1.01 | 1.00, 1.01 | <0.001* |
|  | Female |  | 1.01 | | 1.00, 1.01 | <0.001* |  | 1.01 | 1.01, 1.01 | <0.001* |  | 1.01 | 1.01, 1.01 | <0.001* |
| TG | Total |  | 1.00 | | 1.00, 1.00 | <0.001* |  | 1.00 | 1.00, 1.00 | 0.022* |  | 1.00 | 1.00, 1.00 | 0.005* |
|  | Male |  | 1.00 | | 1.00, 1.01 | 0.002* |  | 1.00 | 1.00, 1.00 | 0.001* |  | 1.00 | 1.00, 1.00 | 0.002* |
|  | Female |  | 1.00 | | 1.00, 1.00 | <0.001* |  | 1.00 | 1.00, 1.00 | 0.082 |  | 1.00 | 1.00, 1.00 | 0.130 |
| TyG | Total |  | 2.49 | | 1.98, 3.15 | <0.001* |  | 1.65 | 1.26, 2.16 | <0.001* |  | 1.91 | 1.42, 2.57 | <0.001* |
|  | Male |  | 1.52 | | 1.10, 2.13 | 0.013* |  | 1.12 | 0.77, 1.65 | 0.500 |  | 1.37 | 0.91, 2.09 | 0.140 |
|  | Female |  | 3.43 | | 2.46, 4.82 | <0.001* |  | 2.33 | 1.59, 3.43 | <0.001* |  | 2.54 | 1.64, 3.94 | <0.001* |
| weighted |  |  |  | |  |  |  |  |  |  |  |  |  |  |
| TC | Total |  | | 1.01 | 1.00, 1.01 | <0.001* |  | 1.01 | 1.00, 1.01 | <0.001* |  | 1.01 | 1.00, 1.01 | 0.003* |
|  | Male |  | | 1.01 | 1.00, 1.01 | <0.001* |  | 1.01 | 1.00, 1.01 | 0.015* |  | 1.01 | 1.00, 1.01 | 0.030* |
|  | Female |  | | 1.01 | 1.01, 1.01 | <0.001* |  | 1.01 | 1.01, 1.01 | <0.001* |  | 1.01 | 1.01,1.01 | 0.001* |
| TG | Total |  | | 1.01 | 1.00, 1.01 | <0.001* |  | 1.00 | 1.00, 1.01 | 0.061 |  | 1.00 | 1.00, 1.01 | 0.100 |
|  | Male |  | | 1.01 | 1.00, 1.01 | <0.001* |  | 1.00 | 1.00, 1.01 | 0.003* |  | 1.00 | 1.00, 1.01 | 0.009* |
|  | Female |  | | 1.00 | 1.00, 1.01 | 0.040* |  | 1.00 | 1.00, 1.01 | 0.400 |  | 1.00 | 1.00, 1.01 | 0.500 |
| TyG | Total |  | | 3.29 | 2.29, 4.71 | <0.001* |  | 1.95 | 1.22, 3.14 | 0.010* |  | 2.32 | 1.32, 4.06 | 0.010* |
|  | Male |  | | 2.12 | 1.21, 3.72 | 0.011* |  | 1.27 | 0.66, 2.48 | 0.400 |  | 1.46 | 0.66, 3.23 | 0.300 |
|  | Female |  | | 4.48 | 3.08, 6.51 | <0.001* |  | 3.04 | 2.01, 4.59 | <0.001* |  | 3.97 | 2.25, 7.02 | <0.001* |

*: *p*<0.05. Model 1: Crude model. Model 2: Adjusted for age, BMI, race, PIR, education level, marital status, alcohol status, smoking status, and physical activity (for total popular gender was added). Model 3: Adjusted for age, BMI, race, PIR, education level, marital status, alcohol status, smoking status, physical activity, cancer, CKD, diabetes, hypertension, and liver disease (for total popular gender was added).
