# Supplementary material for: Associations between estradiol and hyperuricemia and the mediating effects of TC, TG, and TyG: NHANES 2013–2016
Source: Front Endocrinol (Lausanne). 2024 Aug 7;15:1422470. doi: 10.3389/fendo.2024.1422470 (PMC11335549; doi:10.3389/fendo.2024.1422470)
Supplement: Supplementary file 2 [file Table_2.docx]

**Supplementary Table 2** Associations of E2 with UAT

| **Characteristic** |  | **Model1** | | | |  | **Model2** | | |  | **Model3** | | |
| --- | --- | --- | --- | --- | --- | --- | --- | --- | --- | --- | --- | --- | --- |
|  |  | **OR** | | **95% CI** | **p-value** |  | **OR** | **95% CI** | **p-value** |  | **OR** | **95% CI** | **p-value** |
| unweighted |  |  | |  |  |  |  |  |  |  |  |  |  |
| Total |  | 0.95 | | 0.90,1.01 | 0.130 |  | 0.91 | 0.84, 0.98 | 0.016* |  | 0.91 | 0.84, 0.99 | 0.022* |
| Male |  | 1.54 | | 1.15, 2.09 | 0.004* |  | 1.31 | 0.94, 1.83 | 0.120 |  | 1.32 | 0.94, 1.85 | 0.100 |
| Female |  | 0.93 | | 0.87, 0.99 | 0.027* |  | 0.92 | 0.84, 1.01 | 0.088 |  | 0.93 | 0.85, 1.02 | 0.130 |
| weighted |  |  | |  |  |  |  |  |  |  |  |  |  |
| Total |  | | 1.01 | 0.93, 1.10 | 0.700 |  | 0.94 | 0.83, 1.07 | 0.300 |  | 0.95 | 0.82, 1.09 | 0.400 |
| Male |  | 1.74 | | 1.15, 2.63 | 0.011* |  | 1.36 | 0.81, 2.26 | 0.200 |  | 1.37 | 0.79, 2.39 | 0.200 |
| Female |  | 0.99 | | 0.91, 1.07 | 0.800 |  | 0.95 | 0.84, 1.07 | 0.400 |  | 0.96 | 0.84,1.10 | 0.500 |

*: *p*<0.05. Model 1: Crude model. Model 2: Adjusted for age, BMI, race, PIR, education level, marital status, alcohol status, smoking status, and physical activity (for total popular gender was added). Model 3: Adjusted for age, BMI, race, PIR, education level, marital status, alcohol status, smoking status, physical activity, cancer, CKD, diabetes, hypertension, and liver disease (for total popular gender was added).
